# Diabetes in Mexico: cost and management of diabetes and its complications and challenges for health policy

**DOI:** 10.1186/1744-8603-9-3

**Published:** 2013-02-02

**Authors:** Simon Barquera, Ismael Campos-Nonato, Carlos Aguilar-Salinas, Ruy Lopez-Ridaura, Armando Arredondo, Juan Rivera-Dommarco

**Affiliations:** 1 Centro de Investigación en Nutrición y Salud, Instituto Nacional de Salud Pública, Av. Universidad No. 655. Col. Sta. Ma. Ahuacatitlán, Cuernavaca, Mor, CP. 62508, Mexico, Mexico; 2 Instituto Nacional de Ciencias Médicas y Nutrición Salvador Zubirán, México, D.F, Mexico

**Keywords:** Diabetes, Costs, Mexico, Prevalence, Diabetes management

## Abstract

**Background:**

Mexico has been experiencing some of the most rapid shifts ever recorded in dietary and physical activity patterns leading to obesity. Diabetes mellitus has played a crucial role causing nearly 14% of all deaths. We wanted to make a comprehensive study of the role of diabetes in terms of burden of disease, prevalence, cost of diabetes, cost of complications and health policy.

**Method:**

We review the quantitative data that provides evidence of the extent to which the Mexican health economy is affected by the disease and its complications. We then discuss the current situation of diabetes in Mexico with experts in the field.

**Results:**

There was a significant increase in the prevalence of diabetes from 1994 to 2006 with rising direct costs (2006: outpatient USD$ 717,764,787, inpatient USD$ 223,581,099) and indirect costs (2005: USD$ 177,220,390), and rising costs of complications (2010: Retinopathy USD$ 10,323,421; Cardiovascular disease USD$ 12,843,134; Nephropathy USD$ 81,814,501; Neuropathy USD$ 2,760,271; Peripheral vascular disease USD$ 2,042,601). The health policy focused on screening and the creation of self-support groups across the country.

**Conclusions:**

The increasing diabetes mortality and lack of control among diagnosed patients make quality of treatment a major concern in Mexico. The growing prevalence of childhood and adult obesity and the metabolic syndrome suggest that the situation could be even worse in the coming years. The government has reacted strongly with national actions to address the growing burden posed by diabetes. However our research suggests that the prevalence and mortality of diabetes will continue to rise in the future.

## Background

Mexico has been the subject of an epidemiological transition: in two decades, Mexico’s disease profile has transformed from malnutrition, communicable infectious and parasitic diseases to a country dominated by obesity, diabetes and other nutrition-related non-communicable diseases (NR-NCDs) [1-3]. Mexico has experienced some of the most rapid shifts in dietary and physical inactivity patterns--and ultimately obesity--ever recorded [4-7]. Between 1988 and 2006, Mexico’s annual prevalence rate of obesity (body mass index [BMI] ≥ 30 kg/m^2^) increased among adults by approximately 2% per year, the largest increase documented worldwide. From 1980 to 2000 researchers documented an alarming 47% increase in diabetes mellitus mortality rates: in 1980 diabetes mellitus was the ninth cause of mortality and ascended to the third by 1997 [2]. Based on national mortality statistics, after disaggregating cardiovascular disease, diabetes has been the primary cause of death among women and men since 2000 followed by coronary heart disease. In 2009, diabetes was responsible for 77,699 deaths, representing 13.76% of all deaths [8].

## Methods

In this paper we review the quantitative data that provides evidence of the extent to which the Mexican health economy is affected by the disease and its complications. We then examine and discuss the current situation of diabetes in Mexico with experts in the field. Our research does not involve human intervention or collection biological samples. This paper only describes information of secondary databases and studies that have been ed by Ethics, Research and Biosecurity Committees of the National Institute of Public Health.

## Results

### Burden

In México, it was estimated in 2004 that NR-NCDs caused 75% of the total deaths and 68% of total disability-adjusted life years (DALYs). The leading causes of death were ischemic heart disease, diabetes mellitus and cerebrovascular disease. High body mass index (BMI), high blood glucose and alcohol consumption are responsible for a larger burden of disease than other non-communicable disease risks; diabetes mellitus accounted for 9.7% of total deaths, with higher percentages in women (12.1%) than men (9.7%) and 3.5% of total DALYs. High blood glucose and high BMI together accounted for 25.3% of total deaths and 10.1% of total DALYs [9].

The country shows very heterogeneous levels of transition by region, a condition that has been called the polarization of the nutrition/epidemiological transition; the southern region of Mexico, which is less developed, showed an increase of diabetes mortality rates from 1980–2000 of 128% compared to the more developed northern region, where mortality increased only 32.5% [2]. The southern region also faces higher prevalence of undernutrition and infectious diseases making this region the one with the largest burden of ill health in the country [9].

### Sources of information: diabetes registers and national surveys

Although there have been some efforts to develop a national registry of diabetes, this has not been accomplished yet. The prevalence of diabetes and other diseases at the national, regional and state level have been obtained from diverse national surveys (the National Chronic Disease Survey 1994, the National Nutrition Survey II and National Health Survey 2000) which recently have been consolidated into the Mexican National Nutrition Survey (ENSANUT 2006 and 2012) collected every six years. A representative subsample of fasting serum, blood sugar, blood lipids, and other biochemical indicators [10] is obtained from participants 20-years and older. These surveys also collect HbA_1c_ from participants that have been previously diagnosed to evaluate control. It also has a section on access to health services. Data on expenditure on medications for diabetes, high blood pressure and obesity and treatments is collected by the National Income and Expenditure Surveys every two years in Mexico by INEGI [11]. Incidence data has been collected from diverse cohorts such as the Mexico City diabetes study, but how representative it is of the entire country is uncertain [12]. Overall, the health statistical system in Mexico has been recognized as one of high quality, mostly due to the Mexican Health and Nutrition Survey. However, as in many other countries, incidence information is scarce [13].

Finally, together with the launch of the medical specialties systems (UNEMES), there was an effort to establish an information system for diabetes outcomes, especially related to quality of care indicators. However this system is still in its development. Besides this, other local efforts have been developed in the Mexican institute of social security (IMSS) and within the certification of diabetic groups, that also might be a reliable source in the near future [14].

### Prevalence

There was a significant increase in the prevalence of diabetes from 1994 to 2006 (the time frame covered by the National Health Surveys). In the ENEC-1994 survey, the prevalence of diabetes mellitus type 2 was 6.7% (previously diagnosed [PD] 4.6% and newly diagnosed [ND] 2.1% or didn’t know who they had diabetes). In the ENSA-2000 study, the overall prevalence was 7.5% (5.8% previously diagnosed and 1.7% newly diagnosed). In the ENSANUT-2006 survey, the prevalence reached 14.4% (7.3% PD and 7.1% ND) (Table 1) [15]. The prevalence increased in both genders. For women, the prevalence was 6.8%, 7.8% and 13.2% in the 1993, 2000 and 2006 surveys respectively. The corresponding percentages were 6.6%, 7.2% and 15.8% in men.

**Table 1:** Prevalence of Diabetes mellitus

**Survey**	** Prevalence**	**Women/Men**	**Urban/Rural**
1994 (ENEC-1994)	6.7%	6.8%/6.6%	--
2000 (ENSA-2000)	7.5%	7.8%/7.2%	8.2%/5.6%
2006 (ENSANUT-2006)	14.4%	13.2%/15.8%	15.5%/10.4%

In both the ENSA-2000 and ENSANUT-2006 surveys (the only two surveys with representation within rural and urban areas), the prevalence was higher in urban (8.2 vs. 15.5% respectively) populations compared to rural ones (5.6 and 10.4% respectively). The estimated overall prevalence of type 2 diabetes between 1993 and 2006 increased by two-fold (7.4 percentage points [pp]), resulting in a rate of 0.56 pp/year. Considering only the PD cases, the prevalence increased from 4.6% in 1993 to 7.3% in 2006. This is an overall increment of 2.7 pp over a 13 year time period (0.2 pp/year).

There is limited information on the incidence of diabetes available in Mexico; the Mexico City study conducted in a low-income population reported a cumulative incidence of 9.12% and 7.22% in males and females respectively, from 35–64 years of age in a 6.3 year follow-up [16]. A study in Mexican-Americans showed a similar diabetes incidence of 6.5% after a 8-year follow-up, with higher rates for males than females [17].

Diabetes mellitus prevalence has reached 14.4% of the population (representing 7.31 million adults) [18] and at the same time it has become the number one general cause of mortality, with 14% of total deaths; in 2008 a total of 75,572 Mexicans died from this cause (un-adjusted mortality rate 70.8/100,000 inhabitants) [19,20].

### Health care system

The Mexican health care system is formed by diverse public institutions that offer health care services to both the uninsured population (Ministry of Health Medical Services) and salaried workers from the tax-paying formal economy (Mexican Institute of Social Security (IMSS) and the Institute for Social Security and Services for State Workers (ISSSTE)). In 2001, Seguro Popular (People’s Insurance) was created by the federal government as a major effort to protect the uninsured population against steep health care costs. Enrolment in Seguro Popular is voluntary and is not dependent on health status or pre-existing illness. There is no co-payment and contributions are determined solely by ability to pay [15], with a predefined quote based on income deciles (the poorest 4 deciles of income without any payments and from the 5th to the top income deciles a quote of USD $152.00 to USD $834 per family per year) [21]. The rest of the population with purchasing power receives medical attention from the private sector [22]. In its last report from 2010, the Seguro Popular had already enrolled 43.5 millions of previously uninsured Mexicans reaching 88.5% of the final goal of universal coverage. The total contributions from family quotes was USD$ 15.5 million, only covering a 0.2% of the total financial sources that for the year 2010 resulted in USD$ 8,043 million [21]. Diabetes mellitus is among the many diseases covered by this insurance, however this coverage includes mainly ambulatory primary care and urgent care, but major costs of chronic complications such as substitution of renal failure and acute coronary syndrome was not covered in 2010. In 2011, myocardial infarction was added to the catalogue of major diseases covered, but only among adults younger than 60 years old [23].

#### Costs of diabetes and costs of complications

Several estimates have been published about the economic burden imposed to the health system by this condition. In the most recent published report, Avila et al. estimated the total national expenditure on diabetes mellitus, cardiovascular disease and obesity in 2006 came to USD$ 2,869.6 million representing 7% of the national health expenditure and 0.4% of the Gross National Product (GNP). From this amount 73% was financed by the state and 27% by the private sector. A total of 40.7% of this estimate was allocated solely to diabetes mellitus [11,24]. In addition to this estimation, which is based on expenditure rather than costs, many other cost estimates have been published in the last two decades (Table 2).

**Table 2:** Cost of diabetes care in Mexico (in US dollars)

**Source**	**Year**	**Methodology**	**Coverage**	**Direct cost**	**Indirect cost**	**Total cost**
Philips M et al.	1992	Not specified	Nationwide	99,936,000	330,000,000	429,936,000
Villarreal et al.	2000	Not specified	Nationwide	2,618,000	Not specified	Not specified
Barcelo et al.	2003	National databases	Nationwide	1,974,200,000	13,144,100,000	15,118,200,000
Arredondo et al.	2004	Surveys, estimation of the mean cost of a typical case	Nationwide	140,410,816	177,220,390	317,631,206
Zhang et al.	2010	Simulations	Nationwide	4,836,480,000	Not specified	Not specified
Rodriguez et al.	2011	Simulations, estimation of the mean cost of a typical case	IMSSS	452,064,988	Not specified	Not specified
Arredondo et al.	2011	Surveys, estimation of the mean cost of a typical case	Nationwide	343,226	435,200,934	778,427,475

Modified from: Rodríguez Bolaños RA, Reynales Shigematsu LM, Jiménez Ruíz JA, Juárez Márquez SA, Hernández Ávila M. Costos directos de atención médica en pacientes con diabetes mellitus tipo 2 en México: análisis de microcosteo. Rev Panam Salud Publica. 2010;28(6):412–20.

### Direct costs of diabetes

In a report published in 2006, the total cost for Diabetes Mellitus in the country was USD$ 1,164.8 million dollars, this amount includes the concepts described in Table 3[11].

**Table 3:** **Direct costs of diabetes (2006)**[11]

	**Costs in US Dollars**
Outpatient	$717,764,787
Inpatient	$223,581,099
Drugs	$222,904,956
Public Health Programmes	$151,779
Health Administration and Medical Insurances	$473,673

These estimates are higher than the ones reported by Arredondo et al. (2005) using different methodology [22], where the total direct and indirect cost amounted to USD$ 317,631,206 (see III. Discussion). A recent update by this group found total costs for 2010 came to USD$ 343,226,541 reflecting an increase of approximately 8% in a 5-year period [25].

### Indirect cost of diabetes

In 2005 indirect costs were estimated at USD$ 177,220,390 (at an exchange rate corresponding to January 2003). From these costs, a major part was the cost of permanently disabled patients (USD$ 166,693,502), followed by the cost of mortality (USD$ 8,010,360), and the cost of temporarily disabled patients (USD$ 2,516, 528) [22].

### Costs of diabetes complications

The main chronic complications of diabetes are nephropathy, cardiovascular disease, retinopathy, neuropathy and peripheral vascular disease. Total annualized average diabetes costs (without complications), is equivalent to $707 US DLLS. When complications appear, this cost increases by 75% when nephropathy is present, 13% for vascular complications, 3% for neuropathy and 8% for retinopathy.

Based on data from the 2006 health survey and with the use of a predictive model, it is estimated that the 53.8% of people currently living with diabetes will die in the following 20 years. The average life expectancy is 10.9 years (95% CI 10.7-11.2). It is expected that over the next 20 years, 889,443 new cases of patients with heart failure (95% CI 509, 638–1, 269, 248), 2,048,996 with myocardial infarctions (95% CI 1,699,743-2,398,248), 798,188 with strokes (95% 544, 809–1, 051, 568) and 491,236 with lower-limb amputations (95% CI 313, 900–668, 572) will occur if the quality of care has not been improved (Table 4).

**Table 4:** Major diabetes complications and their direct costs in Mexico

	Direct costs attributable to diabetes complications in US dollars*	
	2005 [22]	2010 [25]
Retinopathy	4,968,491	10,323,421
Cardiovascular disease	4,516,810	12,843,134
Nephropathy	32,972,722	81,814,501
Neuropathy	1,626,050	2,760,271
Peripheral vascular disease	1,084,033	2,042,601

* 2005 estimates were for the three main public institutions of the Mexican health care system; 2010 estimates included private costs and private insurances.

Trends revealed by comparing incremental costs for complications from 2005 to 2010 can be explained by three main reasons:

■ Despite promotion and prevention programmes for complications of diabetes, epidemiological incremental changes generated significant increases in demand for care for diabetes complications.

■ Costs from 2005 to 2010 are steadily increasing.

■ Possible changes in the organization of health systems in relation to the combination of inputs to meet the health care required for the five major complications of diabetes.

### Diabetes prevention, screening and treatment and outcome

#### Prevention

Since 2001, there have been explicit national diabetes action programmes and the National Health Plan had an important focus on NCDs [26]. During the 2007–2012 federal administration, NCDs became a top priority in the National Health Plan [27], and efforts in prevention, treatment and control were intensified based on a specific action programme for diabetes mellitus [28]. Some of the important achievements in diabetes prevention and control developed by the Ministry of Health (MOH) and the public health services since 2000 are described in Table 5.

**Table 5:** **Recent government actions to prevent and control diabetes**[27,28]


1.	Development of massive communication programmes to raise awareness of the disease and of the benefits of healthy weight, adequate diet and physical activity.
2.	Regulation of food distributed in Mexican primary schools.
3.	Launch of massive self-care diabetes campaigns.
4.	Unification of guidelines and criteria to diagnose and control diabetes.
5.	Development of self-support groups for diabetic patients.
6.	Strengthening of knowledge and competences for health personnel and improvement of access to information by the health sector and general population.
7.	Development of a National Health Card for adults (similar to the children vaccination card), in which criteria and objectives for health risks are prioritized and evaluations of healthy weight, blood sugar, blood pressure and lipids are emphasized.

One important success in this period was the creation of self-support groups across the country. Currently there are more than 11,000 groups receiving orientation, guidelines and certification from the MOH. Screening has also improved substantially; in 2000, 10% of adults requested this service from the MOH. Six years later, one out of every five adults went to public medical services for blood glucose screening [28].

#### Screening

Currently, screening is based on an opportunistic strategy with sporadic population base campaigns. The MOH established a combined strategy of diabetes and hypertension screening using an adaptation of the ADA questionnaire and strategy in the case of diabetes [29]. However its validity has been considered questionable because of its specificity.

#### Treatment

A recent report found that adequate control is very rare for members of the population that participated in ENSAUT 2006 that were previously diagnosed with diabetes; only 6.6% of those diagnosed had HbA1c <7%. One of the identified reasons for this increase in mortality and lack of control has been the suboptimal efficacy of the current therapeutic model. In addition, this report found that most of the known diabetes population in Mexico are in poor control, regardless of access to care, type of institution, or insurance [30].

##### The poor management of hyperglycemia is not due to lack of access to health services

The majority of patients are under treatment (94.1%), which is based on the use of glucose lowering drugs in most cases (84.8%). However, only a minority understands the importance of life style modifications, e.g. the eating habits (24.1%) and exercise (1.8%) as part of its management.

##### The use of insulin is delayed and it is indicated in a small number of cases (6.8%) compared to international standards (> 20%)

The same phenomenon occurs with other clinical variables that should be modified for the prevention of chronic complications.

##### Half of the people with diabetes have high blood pressure

Among those previously diagnosed cases, 80% received treatment. However, the majority (76.7%) has blood pressure values above the recommended targets (130/85 mmHg). The same phenomenon can be seen with the treatment of dyslipidemia (DM) and the use of antiplatelet drugs.

Other NR-NCDs in Mexico follow the same pattern of DM; obesity is steadily increasing as well as high blood pressure and other NR-NCD mortality causes such as ischemic heart disease. Cardiovascular and liver diseases are on the increase too [7,31]. In terms of insulin use, many potential barriers have been proposed by the attending physician, the patient and the institution itself. However, recent efforts in public institutions are focusing on how to increase insulin use among diabetics [32]. In Mexico, public health services are obliged to provide all prescribed medications to patients if included in the basic medication scheme (a list of generic drugs). When a prescribed medication is not on this list, it must be purchased by direct payment by the patient in a drug store or with charge to the institution, when it is justified according to specific criteria [33].

Some evidence of availability of drugs from the basic medication scheme (Table 6) in primary health services has been documented [34].

**Table 6:** Drugs included in the basic scheme used to treat diabetes


1.	Glyburide, pioglitazone, rosiglitazone, metformin, acarbose.
2.	Intermediate action human insulin NPH
3.	Rapid action regular human insulin
4.	Intermediate-slow action human insulin
4.	Lispro insulin
5.	Lispro protamine

The increasing diabetes mortality and lack of control among diagnosed patients make quality of treatment a major concern in Mexico. The growing prevalence of childhood and adult obesity and the metabolic syndrome suggest that the situation could be even worse in the following years [1,8,35,36]. An analysis of diabetes care in the Mexican population using data from ENSANUT 2006 reported that 25.6% of the previously diagnosed cases did not have access to care; from this percentage 73.4% were females and this group had the highest proportion of subjects speaking an aboriginal dialect and living in rural areas. This study showed that 85.6% of diagnosed patients are treated with oral agents, 6.2% reported no pharmacological therapy and a very small proportion of the population was using insulin (as a single therapeutic agent or in combination). Only a small fraction of the participants had adequate HbA1c levels and the group with no access to health care had similar values of mean HbA1c compared to the rest of the groups. A total of 84% of the population with HbA1c was poorly controlled and more than half of these had levels above 12% [30]. Among the factors that were associated to a better control in this survey were: a medical consultation within the last three months, and access to social security. The participation of a dietitian in the medical attention of the participant decreased the odds of being severely uncontrolled (RM = 61, 95%CI = 0.38-0.97) [37].

There are different sets of guidelines and norms in Mexico, however recently an attempt to unify criteria has been made. The NOM (Mexican Official Norm) NOM-015-SSA2-1994 described the general treatment for diabetes in medical practice. A new norm has been released with more updated criteria in 2010 (NOM-015-SSA2-2010). The UNEMES-Crónicas (previously described), have protocols for interdisciplinary treatment of diabetes mellitus and other diseases [38]. IMSS, ISSSTE and other public health services have their own guidelines. There is also consensus in publications from medical societies promoting recommendations for DM treatment as well as position papers [39,40].

#### Diabetes outcome

Each Mexican health system has his own set of diabetes-related outcomes. However, their databases are not integrated into a national registry. Outcomes are recorded for all hospitalized patients by the attending physicians; data is sent to a central office. However, outcomes are not recorded in the majority of the outpatient clinics (except for the UNEMES chronic units). Process indicators are recorded in some but not in all health systems. Their recollection started in the last five years.

## Discussion

### Weak evidence base due to lack of solid data

#### Data basis

Currently the major registry is within each public institution, especially IMSS and ISSSTE, and its primary use is administrative. Periodically the Minister of Health reports on the number of diabetics registered as new cases and estimates of incidence rates. However, due to the lack of a diabetes registry, these estimates are not often used for epidemiological or administrative purposes. Moreover, since the year 2000, the MOH has started another sentinel surveillance system for hospitalized cases of diabetes but similar to the periodic surveillance reports, the data obtained lacks validity for any systematic use. The last report from this system was in 2007 [41].

In addition to the information system, Mexico has a very well developed system for reporting mortality and prevalence information based on national surveys. In Mexico, date on diabetes mellitus mortality is obtained from the National Mortality Statistics Registry managed by the National Institute of Statistics, Geography and Informatics (INEGI http://www.inegi.org.mx).

#### Prevalence data

There are multiple reasons for the observed changes in the prevalence of diabetes. These include a shift in the age distribution of the population and a growing prevalence of obesity, principally related to changes in lifestyle. However there are several methodological differences between surveys that might explain part of the ascendant trend. The proportion of subjects studied under fasting conditions was small in both the 1993 and 2000 surveys (≈5%). As a result, most of the newly diagnosed cases were identified by the random blood glucose criterion (200 mg/dl). This characteristic of the 1993 and 2000 studies may have led to an underestimation of the true prevalence; random glucose measurements are the diagnostic criterion with the lowest sensitivity. This was not the case for the ENSANUT-2006 survey. Fasting was verified in all participants of the subsample in which the prevalence of diabetes was estimated.

Another indicator of a possible underestimation of the type 2 diabetes prevalence in the two earlier surveys is the proportion of previously diagnosed to newly diagnosed cases. This ratio was 1:0.45 for ENEC-93, 1:0.26 for ENSA-2000 and 1:0.97 for ENSANUT-2006 respectively. The PD:ND ratio in the National Health and Nutrition Examination Survey of North America (NHANES-III) was 1:0.5 [21]. The high prevalence of diabetes mellitus is associated with an earlier age of onset in the majority of the population [9]. The prevalence of type 2 diabetes diagnosed before the age of 40 increased progressively from 1.8% (PD = 0.95%, ND = 0.65%) in 1993 to 2.3% (PD = 1.19%, ND = 1.51%) in 2000 and to 5.7% (PD = 1.45%, ND = 4.26%) in 2006. The surge of early onset diabetes seen in ENSANUT-2006 corresponded predominantly to cases diagnosed during the survey.

Individuals diagnosed before the age of 40 will have a longer exposure to hyperglycemia and other diabetes-related abnormalities, ultimately increasing the likelihood of chronic complications. Also, this type 2 diabetes will require insulin therapy early on. Studying this variant of the disease will render strategic information for health care planning in Mexico; detection campaigns and preventive actions have to be targeted to subjects younger and older than 40 years. However, this strategy has to be proven to be cost effective in order to establish it as a public policy.

#### Costs of diabetes – methods

Large differences exist between reports on costs; inconsistencies arise due to the sources of information used and the assumptions and models applied. Depending on the study referred to, these differences can be further explained by one or more of the following reasons:

■ The population base and the method of costing may be based only on estimates and probabilistic simulations without information on actual costs or actual cases from national health system.

■ The population base and the method of costing may be based on actual costs and actual cases by type of institution for the entire national health system.

■ The concept of costs and expenses are different; some studies are not explicit or clear in regards to expenditure or cost depending on the sources of information and the type of inputs and costs.

■ In the case of the methodology based on annual average cases, differences in the amounts are explained by differences in the type and quantity of inputs used by each institution depending on the production function in question and the quality standards attention as intervening variable in the control or management cost average. This kind of estimate is more related to the “ideal” cost, rather than the actual cost. For instance, the differences in the total amounts of direct and indirect costs can also be explained by differences in the sources of cost information. Furthermore, the cost of inputs varies depending on social security institutions, for public assistance and private institutions and users.

#### Treatment

The lack of effectiveness of treatment is explained by factors attributable to the health system, the doctor and the patient. Although Mexican guidelines for the treatment of diabetes exist, few doctors are familiar with them or apply them. In addition, primary care clinics (responsible for the treatment of the majority of the cases) do not have the infrastructure to treat chronic diseases. Diabetes management involves a learning process to understand the disease, the changes needed in behavior, the use of multiple drugs, the frequency of evaluations as well as the participation of specialists in conjunction with the family and the community. However, the current organizational structure and procedures of the majority of health institutions are not prepared to provide it.

Visits between doctor and patient are seldom programmed. The duration of individual consultations is insufficient and the inclusion and participation of other health professionals only occurs in a few instances.

At present, the training of health professionals does not adequately prepare them for the realities of practice. Educational programmes provide a priority to combat degenerative diseases in the medium term; however, many graduates have the knowledge but lack the skills to promote an effective treatment. As a result, clinical inertia is a major contributor to the inefficiency of the system.

Finally, public awareness of the disease is low. Patients often do not understand the treatment goals and do not make the necessary lifestyle changes.

Programmes to improve the quality of diabetes treatment have been presented but poorly implemented. The lack of infrastructure of the teams in charge of the programmes is the main reason for the lack of success.

## Conclusions - health policy

The government has reacted strongly with national initiatives to address the growing burden posed by diabetes. However our research suggests that the prevalence and mortality of diabetes will continue to rise in the coming years. A national programme for adult and elderly health integrates efforts for diabetes prevention, education and control at the federal Ministry of Health. In addition, the federal administration launched a system of primary health clinics focused on the treatment of obesity, diabetes mellitus, dyslipidemias and high-blood pressure (UNEMES cronicas), with an interdisciplinary team (comprised of a psychologist, social worker, dietitian, nurse and physician) [42]. These clinics developed improved guidelines for prevention, detection and control of NCDs with an important behavioural component. They also are using technology to optimize attention and improve monitoring and evaluation.

This model is based on experiences in Ireland, London, the US and Europe [43-46]. More than 50 clinics are now operating in most states of the country. Information and evaluations of this pilot programme would be useful to quantify the potential impact of these initiatives. These clinics are able to treat 3,000 patients/year. There is at least one of the almost 100 UNEMES specialized health clinics in the majority of states in Mexico.

There are additional government initiatives that set out to complement the diabetes programme. Primary care physicians and members of the “UNEMES cronicas” teams are trained in virtual courses (diploma) in chronic diseases (including evaluation and prescription of diet and physical activity, obesity, diabetes, dyslipidemias and high blood pressure treatment and adherence, and motivational interviewing training coordinated by the National Institute of Public Health for approximately 2,000 health professionals).

The MOH created a National Council for the Prevention and Control of Chronic Diseases and promoted a nation-wide communication programme called “Five Steps for Your Health” (“Cinco pasos por tu salud”). This programme included recommendations to consume water (instead of soft drinks or other caloric beverages), increase physical activity, increase consumption of fruits and vegetables, and regular weight checks. A total of six television advertisements on 440 television channels and 750 radio stations are broadcasted daily promoting this programme which reaches an audience of approximately 70 million people [47].

During 2010, The president of Mexico announced that the MOH together with the National Institute of Public Health had developed the first national policy to prevent obesity and promote healthy nutrition [48]. In addition to these federal efforts, there are several state programmes to prevent diabetes. IMSS, ISSSTE and other public health services have developed their own national programmes based on common guidelines. In a propensity score matching analysis, poor diabetic patients registered with Seguro Popular (part of the social health protection system) showed in a preliminary analysis that they had improved access to health care and blood glucose control [15].

## Recommendations: priorities for the future

1. There is an urgent need to obtain evaluations of the current preventive and control actions taking place in the country.

2. More resources must be directed to diabetes prevention and on translational research; currently it represents only a minor amount of the total diabetes costs.

3. Multidisciplinary teams should be available in every primary care unit.

4. Primary care physicians should be re-trained to improve their abilities to manage chronic diseases. Certification programmes are urgently needed for the diabetes related competences. Additional internists, endocrinologists and other specialists involved in diabetes care should be incorporated into the various Mexican health systems, although a focus on primary care should be the top priority.

5. Programmes inmedical schools should focus on improving the capabilities of their students to treat chronic diseases.

6. Health systems should be re-organized to improve the quality of the services provided. Certification programmes for the diabetes units are urgently needed. The use of electronic records (using the same process indicators and outcomes) should be implemented in all health systems.

7. Programmes designed to improve diabetes care in Mexico should be coordinated by a full-time dedicated multidisciplinary team with enough resources to implement the required changes.

8. A diabetes national registry should be created.

9. Research and training for the adequate treatment and control of diabetes should be increased in these key areas: a) behavior modification, adherence and motivation; b) nutrition and physical activity evaluation, prescription and monitoring; and c) evaluation, monitoring and pharmacological treatment for health professionals.

10. There should be more regulations promoting a healthy environment to facilitate the adoption of healthy lifestyles:

■ regulate food and beverage marketing to children and adolescents;

■ provide incentives for increasing consumption of fruits and vegetables;

■ develop a government food and beverage front-of-pack labeling system to promote healthier choices among the population and incentivize the food industry to reformulate and develop healthier products;

■ invest in nutrition education and physical activity promotion; and

■ promote water consumption as the preferred hydration alternative.

11. Information systems need to be improved in order to obtain precise incidence information.

12. Laboratory systems should be upgraded for the adequate monitoring of patients.

13. New therapeutic drugs including insulin should be made more accessible within the primary level of care.

## Competing interests

In the past five years have you received reimbursements, fees, funding, or salary from an organization that may in any way gain or lose financially from the publication of this manuscript, either now or in the future? Is such an organization financing this manuscript (including the article-processing charge)? If so, please specify.

## Authors’ contribution

SB Designed the structure of the paper, analyzed the information and wrote the paper with co-authors. ICN Analyzed the information, reviewed the findings and wrote the paper with co-authors. CAS Discussed the preliminary data, reviewed the findings and revised the manuscript. RLR Analyzed the information and wrote the paper with co-authors. AA Analyzed the information and wrote the paper with co-authors. JRD Reviewed the findings and revised the manuscript. All authors read and approved the final manuscript.

## Authors’ information

SB is Director of the Area of Nutrition Policies and Programmes, in the National Institute of Public Health. He has not received any fees, reimbursement or honorary in relation to this work.

ICN is Research of the Research Unit in Chronic Diseases and Diet, in the National Institute of Public Health. He has not received any fees, reimbursement or honorary in relation to this work.

CAS is Research of the Department of Endocrinology and Metabolism, in the National Institute of Medical Sciences and Nutrition-Salvador Zubiran. He has not received any fees, reimbursement or honorary in relation to this work.

RLR is Research of Center for Population Health: Chronic Disease Division, in the National Institute of Public Health. He has not received any fees, reimbursement or honorary in relation to this work.

AA is Research of Center for Research in Health Systems, in the National Institute of Public Health. He has not received any fees, reimbursement or honorary in relation to this work.

JRD is Director of Center of Research in Nutrition and Health in the National Institute of Public Health. He has not received any fees, reimbursement or honorary in relation to this work.

## References

[B1] Rivera JA Barquera S Campirano F Campos I Safdie M Tovar V Epidemiological and nutritional transition in Mexico: rapid increase of non-communicable chronic diseases and obesity Public Health Nutr 2002 5 1a 113 122 12027273 10.1079/PHN2001282

[B2] Barquera S Tovar-Guzman V Campos-Nonato I Gonzalez-Villalpando C Rivera-Dommarco J Geography of diabetes mellitus mortality in Mexico: an epidemiologic transition analysis Arch Med Res 2003 34 5 407 414 10.1016/S0188-4409(03)00075-4 14602508

[B3] Barquera S Hotz C Rivera JA Tolentino ML Espinosa J Campos I Shamah T Kennedy G, Nantel G, Shetty P Food consumption, food expenditure, anthropometric status and nutrition-related diseases in Mexico The double burden of malnutrition: case studies from six developing countries 2006 Rome: Food and Agriculture Organization of the United Nations 161 204

[B4] Popkin BM Understanding global nutrition dynamics as a step towards controlling cancer incidence Nat Rev Cancer 2007 7 1 61 67 10.1038/nrc2029 10.1038/nrc2029 17186019

[B5] Barquera S Campirano F Bonvecchio A Hernandez-Barrera L Rivera J Popkin B Caloric beverage consumption patterns in Mexican children Nutr J 2010 9 1 47 10.1186/1475-2891-9-47 20964842 PMC2987771

[B6] Barquera S Hernandez-Barrera L Tolentino ML Espinosa J Ng SW Rivera JA Popkin BM Energy intake from beverages is increasing among Mexican adolescents and adults J Nutr 2008 138 12 2454 2461 10.3945/jn.108.092163 19022972

[B7] Barquera S Campos-Nonato I Hernández-Barrera L Flores M Durazo-Arvizu R Kanter R Rivera JA Obesity and central adiposity in Mexican adults: results from the Mexican National Health and Nutrition Survey 2006 Salud Publica Mex 2009 51 S595 S603 20464235 10.1590/s0036-36342009001000014

[B8] Rull JA Aguilar-Salinas CA Rojas R Rios-Torres JM Gómez-Pérez FJ Olaiz G Epidemiology of type 2 diabetes in Mexico Arch Med Res 2005 36 3 188 196 10.1016/j.arcmed.2005.01.006 15925009

[B9] Villalpando S de la Cruz V Rojas R Shamah-Levy T Ávila MA Gaona B Rebollar R Hernandez L Prevalence and distribution of type 2 diabetes mellitus in Mexican adult population: a probabilistic survey Salud Publica Mex 2010 52 S19 S26 20585724 10.1590/s0036-36342010000700005

[B10] Veláquez M Lara-Esqueda A Martínez M Márquez F La detecció n integrada como un instrumento para vincular la prevenció n primaria, el tratamiento temprano, y la vigilancia epidemioló gica en diabetes e hipertensió n arterial Rev Endoc Nutr 2000 8 4 129 135

[B11] Burke JP Williams K Haffner SM Villalpando CG Stern MP Elevated incidence of type 2 diabetes in San Antonio, Texas, compared with that of Mexico City, Mexico Diabetes Care 2001 24 9 1573 1578 10.2337/diacare.24.9.1573 11522701

[B12] Barquera S Campos-Nonato I Hernández-Barrera L Villalpando S Rodríguez-Gilabert C Durazo-Arvizú R Aguilar-Salinas CA Hypertension in Mexican adults: results from the National Health and Nutrition Survey 2006 Salud Publica Mex 2010 52 S63 S71 20585731 10.1590/s0036-36342010000700010

[B13] Lerman I Barreras que dificultan la aplicació n temprana de insulina en el paciente con diabetes tipo 2, Volume 7 2009 2 México: Asociación Latinoamericana de Diabetes 66 68

[B14] Leyva-Flores R Erviti-Erice J Kageyama-Escobar ML Arredondo A Prescripción, acceso y gasto en medicamentos entre usuarios de servicios de salud en México Salud Publica Mex 1998 40 24 31 9567655

[B15] Stevens G Dias RH Thomas KJA Rivera JA Carvalho N Barquera S Hill K Ezzati M Characterizing the epidemiological transition in Mexico: national and subnational burden of diseases, injuries, and risk factors PLoS Med 2008 5 6 e125 10.1371/journal.pmed.0050125 18563960 PMC2429945

[B16] Barquera S Tolentino ML Chapela M La obesidad y la diabetes en México: problemas de salud pública en aumento En el debate: Diabetes en México 2010 Mexico DF: Universidad Autónoma Metropolitana 53 84

[B17] Durazo-Arvizu R Barquera S Franco M Lazo M Seuc A Orduñez P Palloni A Cooper R Cardiovascular diseases mortality in Cuba, Mexico, Puerto Rico and US Hispanic populations Prev Control 2006 2 2 63 71 10.1016/j.precon.2006.10.004

[B18] Reynoso-Noveron N Mehta R Almeda-Valdes P Rojas-Martinez R Villalpando S Hernandez-Avila M Aguilar-Salinas CA Estimated incidence of cardiovascular complications related to type 2 diabetes in Mexico using the UKPDS outcome model and a population-based survey Cardiovasc Diabetol 2011 10 1 10.1186/1475-2840-10-1 21214916 PMC3023678

[B19] Secretaría de Salud: Programa de Acción Diabetes Mellitus 2001 Mexico DF: Secretaría de Salud

[B20] Secretaría de Salud: Programa Nacional de Salud 2007–2012 Por un Mé xico sano: construyendo alianzas para una mejor salud 2007 Mexico DF: Secretaría de Salud

[B21] Barquera S Campos-Nonato I Carrión-Rábago C Villalpando S López-Ridaura R Rojas R Aguilar-Salinas CA Methodology for the analysis of type 2 diabetes, metabolic syndrome and cardiovascular disease risk indicators in the ENSANUT 2006 Salud Publica Mex 2010 52 Suppl 1 S4 S10 20585728 10.1590/s0036-36342010000700003

[B22] Gonzalez C Stern MP Gonzalez E Rivera D Simon J Islas S Haffner S The Mexico City diabetes study: a population-based approach to the study of genetic and environmental interactions in the pathogenesis of obesity and diabetes Nutr Rev 1999 57 5 71 77 10391030 10.1111/j.1753-4887.1999.tb01792.x

[B23] Tobias M Subnational burden of disease studies: Mexico leads the way PLoS Med 2008 5 6 e138 10.1371/journal.pmed.0050138 18563966 PMC2429946

[B24] Haffner SM Hazuda HP Mitchell BD Patterson JK Stern MP Increased incidence of Type II Diabetes Mellitus in Mexican Americans Diabetes Care 1991 14 2 102 108 10.2337/diacare.14.2.102 2060411

[B25] Gómez-Dantés O Garrido-Latorre F Tirado-Gómez LL Ramírez D Macías C Abastecimiento de medicamentos en unidades de primer nivel de atención de la Secretaría de Salud de México Salud Publica Mex 2001 43 224 232 10.1590/S0036-36342001000300008 11452699

[B26] Rojas R Aguilar-Salinas CA Jiménez-Corona A Shamah-Levy T Rauda J Avila-Burgos L Villapando S Lazcano-Ponce E Metabolic syndrome in Mexican adults: results from the National Health and Nutrition Survey 2006 Salud Publica Mex 2010 52 Suppl 1 S11 S18 20585723 10.1590/s0036-36342010000700004

[B27] Hernandez-Romieu AC Elnecave-Olaiz A Huerta-Uribe N Reynoso-Noveron N Analisis de una encuesta poblacional para determinar los factores asociados al control de la diabetes mellitus en Mexico Salud Publica Mex 2011 53 1 34 39 10.1590/S0036-36342011000100006 21340138

[B28] Secretaría de Salud: Diabetes mellitusprotocolo para tratamiento en las UNEMES de enfermedades crónicas 2010 México DF: Secretaría de Salud

[B29] Aguilar-Salinas CA Gómez-Pérez FJ Declaración de Acapulco: propuesta para la reducción de la incidencia de la diabetes en México Rev Inves Clín 2006 58 71 77 16789601

[B30] Aguilar-Salinas C Rull J García E Zúñiga S Vázquez C Palacios A Consenso Mexicano para la prevención de las complicaciones crónicas de la diabetes tipo 2: avalado por la Sociedad Mexicana de Nutrición y Endocrinología, Asociación de Medicina Interna de México y la Sociedad de Nutriología Rev Inves Clin 2010 52 325 363 11203107

[B31] SSA: Diabetes mellitus Subsecretaria de Prevención y Promoción de la Salud 2007 1 México: Secretaria de Salud, Primera edición 1 74

[B32] Jimenez-Corona A Rojas R Gomez-Perez FJ Aguilar-Salinas CA Early-onset type 2 diabetes in a Mexican survey: results from the National Health and Nutrition Survey 2006 Salud Publica Mex 2010 52 Suppl 1 S27 S35 20585726 10.1590/s0036-36342010000700006

[B33] Córdova-Villalobos JA Barriguete-Meléndez JA Lara-Esqueda A Barquera S Rosas-Peralta M Hernández-Avila M De Léon-May ME Aguilar-Salinas CA Las enfermedades crónicas no transmisibles en México: sinopsis epidemiológica y prevención integral Salud Publica Mex 2008 50 5 419 427 10.1590/S0036-36342008000500015 18852939

[B34] Landon BE Hicks LS O’Malley AJ Lieu TA Keegan T McNeil BJ Guadagnoli E Improving the management of chronic disease at community health centers N Engl J Med 2007 356 9 921 934 10.1056/NEJMsa062860 17329699

[B35] González-Villalpando C López-Ridaura R Campuzano JC González-Villalpando ME The status of diabetes care in Mexican population: are we making a difference? Results of the National Health and Nutrition Survey 2006 Salud Publica Mex 2010 52 S36 S43 20585727 10.1590/s0036-36342010000700007

[B36] Renders CM Valk GD Griffin SJ Wagner EH van Eijk JT Assendelft WJJ Interventions to improve the management of diabetes in primary care, outpatient, and community settings Diabetes Care 2001 24 10 1821 1833 10.2337/diacare.24.10.1821 11574449

[B37] See Tai S Nazareth I Donegan C Haines A Evaluation of general practice computer templates lessons from a pilot randomised controlled trial Meth Inf Med 1999 38 177 181 10522120

[B38] Smith S Bury G O’Leary M Shannon W Tynan A Staines A Thompson C The North Dublin randomized controlled trial of structured diabetes shared care Fam Pract 2004 21 1 39 45 10.1093/fampra/cmh109 14760042

[B39] Secretaría de Salud Documento base. Cinco pasos por tu Salud 2011 Mexico DF: Secretaría de Salud

[B40] Secretaría de Salud Acuerdo nacional para la salud alimentaria: estrategia contra el sobrepeso y la obesidad 2010 Mexico DF: Sub-Secretaría de Prevención y Promoción de la Salud de la Secretaría de Salud

[B41] Secretaría de Salud: Programa de Acción Específico 2007–2012 Diabetes Mellitus 2007 Mexico DF: Secretaría de Salud

[B42] Sosa-Rubí SG Galárraga O López-Ridaura R Diabetes treatment and control: the effect of public health insurance for the poor in Mexico Bull World Health Organ 2009 87 512 519 10.2471/BLT.08.053256 19649365 PMC2704037

[B43] SSA: Sistema de protección social en salud Informe de resultados: Secretaria de Salud-Seguro Popular 2010 1 México: Primera edición 1 93

[B44] Arredondo A Zúñiga A Economic consequences of epidemiological changes in diabetes in middle-income countries Diabetes Care 2004 27 1 104 109 10.2337/diacare.27.1.104 14693974

[B45] SSA: Sistema de protección social en salud Informe de resultados Enero-Junio: Secretaria de Salud-Seguro Popular 2011 3 México: Tercera edición 1 88

[B46] Avila Burgos L Cahuana Hurtado L Gonzalez Dominguez D Centro de Investigació n en Sistemas de Salud (Mexico): Cuentas en diabetes mellitus, enfermedades cardiovasculares y obesidad: Mé xico 2006 2009 1 Cuernavaca, Morelos, Mé xico: Instituto Nacional de Salud Pú blica

[B47] Centro de Estudios de las Finanzas Públicas de la Cámara de Diputados Average exchange rates Mexican Peso- US Dollar 1970–2011 2011 Mexico DF: Camara de Diputados

[B48] Arredondo A Recaman AL De Icaza E Chapela M Indicadores de evaluación económica de la diabetes en México: implicaciones para el sistema de salud y la sociedad En el debate: Diabetes en México 2010 Mexico DF: Universidad Autónoma Metropolitana 85 110

